# Revisiting water-in-salt electrolytes with ionic liquid incorporation through high-resolution liquid-structure mapping

**DOI:** 10.1093/nsr/nwag239

**Published:** 2026-04-22

**Authors:** Zefang Yang, Xin Liu, Dongyuan Zhao, Dongliang Chao

**Affiliations:** Laboratory of Advanced Materials, Aqueous Battery Center, State Key Laboratory of Molecular Engineering of Polymers, College of Smart Materials and Future Energy, Fudan University, China; School of Physical and Mathematical Sciences, Nanyang Technological University, Singapore; Laboratory of Advanced Materials, Aqueous Battery Center, State Key Laboratory of Molecular Engineering of Polymers, College of Smart Materials and Future Energy, Fudan University, China; School of Electrical and Electronic Engineering, Harbin University of Science and Technology, China; Laboratory of Advanced Materials, Aqueous Battery Center, State Key Laboratory of Molecular Engineering of Polymers, College of Smart Materials and Future Energy, Fudan University, China; Laboratory of Advanced Materials, Aqueous Battery Center, State Key Laboratory of Molecular Engineering of Polymers, College of Smart Materials and Future Energy, Fudan University, China

The water-in-salt (WiS) strategy has fundamentally shaped our understanding of aqueous electrolytes by reducing water activity through ultrahigh salt concentrations [1]. The introduction of lithium bis(trifluoromethanesulfonyl)imide, which exhibits a solubility exceeding 20 m in water, enabled the electrochemical stability window (ESW) of the aqueous electrolyte to expand from the conventional value of ∼1.23 V to nearly 3 V. This advancement has opened new opportunities for high-voltage aqueous batteries [2]. The essence of the WiS concept is to suppress gas evolution, interfacial corrosion, and structural instability by minimizing the activity of bulk-like water. Meanwhile, WiS electrolytes further stabilize electrode reactions through the involvement of anions in solvation shell and interfacial film formation [3]. Nevertheless, in zinc-based systems, the range of electrolytes capable of achieving the WiS regime remains limited, primarily due to the insufficient solubility of most common zinc salts in water [4].

**Figure 1.**
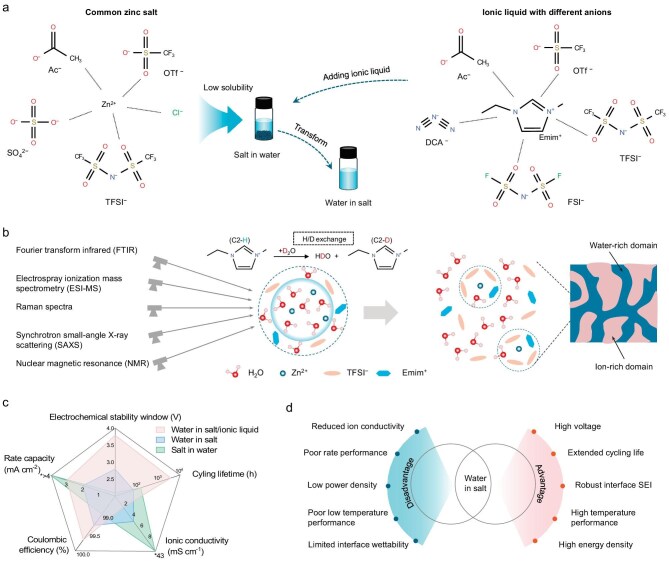
(a) Schematic of the transformation of a low-solubility zinc salt into Wi(S/IL) through the addition of an ionic liquid. (b) The liquid structure of Zn(TFSI)_2_–EmimTFSI–H_2_O ternary electrolyte revealed by multiscale characterization techniques. (c) Radar plot of electrochemical properties of zinc anode using salt in water, water in salt, and water in salt/ionic liquid electrolytes. (d) A comparison of the advantages and disadvantages of the WiS strategy in aqueous batteries.

To address this issue, Chen and co-workers recently reported, in *National Science Review*, a water-in-(salt/ionic liquid) [Wi(S/IL)] design framework that incorporates ionic liquids to expand the compositional space of high-concentration zinc electrolytes and clarify their complex liquid structures [5]. The authors systematically combined five common zinc salts with five commercial ionic liquids, yielding 25 zinc salt–IL–H_2_O systems (Fig. 1a). Among these, 16 systems formed single-phase liquid Wi(S/IL) electrolytes, demonstrating the general applicability of this strategy. On this basis, Zn(TFSI)_2_–EmimTFSI–H_2_O was selected as a model system because of its large single-phase liquid region and broad compositional tunability.

A long-standing question in WiS concerns how water escapes from the first solvation shell of Zn^2+^ and redistributes across both local and long-range scales once its reactivity is significantly suppressed. To probe this process, the authors dissected the local environment of water through spontaneous H/D exchange between D_2_O and the C2–H bond of the Emim^+^ cation, employing the O–D vibration of HOD molecules as a sensitive spectroscopic marker. Combining nuclear magnetic resonance, Fourier-transform infrared spectroscopy, Raman, electrospray ionization mass spectrometry and synchrotron synchrotron small-angle X-ray scattering, they revealed that the first solvation shell of Zn^2+^ is dominated by weakly coordinated anions (Fig. 1b). Water molecules expelled from the solvation shell are not randomly dispersed; instead, they reorganize through interactions with Emim^+^ and TFSI^−^ to form hydrogen-bonded networks and nano-segregated heterostructures comprising distinct water-rich and ion-rich domains. This confinement of free water fundamentally restructures the liquid microenvironment, thereby redefining reaction pathways and governing electrochemical kinetics [6].

This liquid structure reorganization leads to markedly improved electrochemical performance. The optimized Wi(S/IL) electrolyte exhibits an ESW of ∼3.8 V, an average Coulombic efficiency of 99.7% in zinc symmetric cells, and excellent long-term cycling stability (Fig. 1c). These improvements are attributed to the outward redistribution of bulk-like water to the outer region of the solvation shell, coupled with the formation of a stable solid-electrolyte interphase (SEI). Although high-concentration electrolytes provide a wider ESW, improved interfacial stability, and the potential for higher energy density, they are also generally accompanied by increased viscosity, reduced conductivity, poor wettability, limited low-temperature performance, and compromised rate and power capability (Fig. 1d). Therefore, WiS should be understood within a design framework that balances interfacial stability and bulk transport, rather than one aimed solely at maximizing voltage [7].

Although the WiS strategy has advanced aqueous batteries considerably, several key aspects should be more carefully considered in future studies aimed at practical deployment. (i) A renewed emphasis on low-cost and high-solubility salts is required. Many high-performance WiS systems still rely on costly salts such as TFSI and FSI, thereby limiting large-scale applicability. In contrast, traditional zinc salts such as ZnCl_2_ and Zn(Ac)_2_ offer practical potential for Zn-WiS systems because of their high intrinsic solubility, despite challenges such as corrosion and limited material compatibility. However, the overall performance of the battery depends not on the electrolyte regulation alone, but on the integrated design of current collectors, separators and interfacial protective layers [8]. (ii) Evaluation of interface stability under long-term operating conditions. It is widely accepted that high-concentration environments facilitate the formation of robust SEI, thereby suppressing water-induced side reactions and enhancing cycling stability. However, most existing evidence comes from ex-situ characterization or tests conducted under relatively mild conditions [9]. Whether these interphases undergo continuous restructuring under harsher conditions, such as long-term cycling, self-discharge, temperature fluctuations, remains largely unresolved. (iii) Assessment of devices under practically relevant conditions. The performance evaluation in laboratory-scale coin cells is insufficient to support the practical implementation of WiS. Instead, realistic conditions involving high mass loading, controlled electrolyte volume, self-discharge, packaging compatibility, long-term storage, gas evolution, and large-scale safety must be considered [10]. In particular, the high-voltage advantages of WiS must be re-evaluated in pouch cells or larger formats, where uneven current distribution, polarization accumulation, and gas evolution risks are often amplified. (iv) Decoupling electrolyte design between the interface and the bulk phase. WiS broadens the ESW by restructuring local solvation environments and inducing the formation of an interphase. However, driving the bulk electrolyte to extremely high concentrations often improves interfacial stability at the expense of rate capability and power density because of increased viscosity and hindered ion transport. An ideal WiS system should simultaneously provide favorable solvation and interfacial structures while maintaining efficient ion transport in the bulk phase, as exemplified by localized WiS and hybrid electrolyte systems.
